# Measurement of functional and morphodynamic neutrophil phenotypes in systemic inflammation and sepsis

**DOI:** 10.1186/s13054-016-1391-5

**Published:** 2016-08-24

**Authors:** Rens Zonneveld, G. Molema, Frans B. Plötz

**Affiliations:** 1 Department of Pediatrics and Scientific Research Center Suriname, Academic Hospital Paramaribo, Paramaribo, Suriname; 2 Department of Pathology & Medical Biology, University Medical Center, Groningen, University of Groningen, Groningen, The Netherlands; 3 Department of Pediatrics, Tergooi Hospitals, Blaricum, The Netherlands

We read with great interest the review by Leliefeld et al. [1] published in *Critical Care* on the role of neutrophils in immune paralysis during systemic inflammation (SI). This is of high clinical importance since immune paralysis potentially increases susceptibility to new infections or may cause inability to clear existing infections, leading to detrimental outcome. One mechanism proposed for immune paralysis is the release of neutrophil populations with decreased microbicidal properties [1]. During SI, heterogeneous subsets of neutrophils exist with different priming states and functions [2]. In particular, large numbers of immature neutrophils appear in the circulation with diminished expression of receptors, important for pathogen killing [3]. We recently reviewed the literature on morphodynamic changes in neutrophils during sepsis [4]. Sepsis increases neutrophil circulating numbers (and percentages of immature neutrophils) and their cell size and stiffness, and decreases migration/chemotaxis, compared with nondiseased conditions or mild infection. Septic neutrophils are also prone to produce neutrophil extracellular traps (NETs).

Both reviews [1, 4] outline shifting neutrophil identity from an innate responder to a complex immune cell with different functional and morphodynamic phenotypes depending on the underlying pathology. The relationship between changes in functional and morphodynamic phenotypes is not completely understood. For example, the inability of circulating neutrophils to migrate to infectious sites during SI may be the result of diminished expression of chemotactic receptors (e.g., CXCR2), which is most profound in newly released immature granulocytes [1]. Others have suggested a hyperadhesive state of the endothelium, due to overexpression of integrins (e.g., CD11b/CD18) on their membrane, thereby decreasing their ability to migrate [5]. Whether this impaired migration interferes with pathogen killing capacity needs to be investigated.

It is important to know that detecting morphodynamic changes in neutrophils is nowadays clinically feasible with novel technologies [4]. Automated hematology analyzers can measure (immature) neutrophil counts and cell sizes, while microfluidic devices can measure migration velocities. These methods represent a diagnostic toolbox that enables multiparameter analysis of neutrophils during SI and sepsis. When combined with technologies that are still in their infancy (e.g., flow cytometry to measure degranulation), bedside measurement of functions of neutrophils and in-vitro studies as proposed by Leliefeld et al. [1] (e.g., measurement of intracellular or extracellular killing activity) will become feasible. This approach will combine functional and morphodynamic neutrophil phenotypes into clinical settings for better understanding of the exact role of neutrophils in SI and sepsis. Ultimately, this may help to determine whether neutrophils can be considered rational targets for therapy.
