# Supplementary material for: Measurement of functional and morphodynamic neutrophil phenotypes in systemic inflammation and sepsis
Source: Crit Care. 2016 Aug 24;20(1):235. doi: 10.1186/s13054-016-1391-5 (PMC4995806; doi:10.1186/s13054-016-1391-5)
Supplement: Related texts

# Reply: Authors’ response

**Authors:** Pieter Leliefeld, Luke Leenen, Janesh Pillay, Leo Koenderman

Numerous studies have focused on the diagnosis and quantification of the innate immune response by measurement of soluble markers (e.g. cytokines, chemokines, bioactive lipids, etc.) in peripheral blood or other body fluids [6]. Particularly, the definition of markers which predict the course of inflammatory complications (e.g., organ failure or secondary infections) induced by trauma and sepsis has gained much attention [7]. Unfortunately, many of these predictive studies failed, merely showing increases in mediators during the clinically most severe period [8]. These measurements therefore lack relevance as predictive markers.

The failure of the abovementioned studies can be explained by the fact that the presence and/or bioavailability of these soluble mediators is affected by many biological processes including differences in clearance rate, binding to natural antagonists, dominant activity of other immunomodulatory mediators, and decoy receptors [8]. There is therefore an increasing interest in utilization of changes in the cellular phenotype of inflammatory cells as a novel diagnostic tool [9], as also discussed by Zonneveld et al. in their letter. This approach assumes that different inflammatory cells integrate all of the proinflammatory and anti-inflammatory signals and change their activation state/phenotype accordingly [9].

A major drawback in the implementation of cellular predictive diagnostics for inflammatory and infectious diseases was the fact that flow cytometry is very labor intensive and difficult to standardize. Recently, the first fully automated flow cytometer, AQUIOS by Beckman Coulter, was developed and is now commercially available [10]. The machine is able to analyze leukocytes in a sample of whole blood in only 20 minutes. In addition, the obtained data can be exported directly to the electronic hospital file system. This allows cellular diagnostics in a 24/7 context with a high reproducibility, as no human action is necessary other than inserting a standardized blood tube in the machine. Preliminary data show that activation of neutrophils in severely injured patients can be monitored by the expression levels of L-selectin (CD62L) and Mac-1 (CD11b) (data not shown).

In conclusion, the concerns raised by Zonneveld et al. for the implementation of cellular diagnostics for inflammatory diseases can be dealt with by application of completely automated flow cytometry in a 24/7 context. We agree that a bedside point-of-care type of analysis is still not possible. However, a report on the status of the innate immune response within 20 minutes is a major step in the immune monitoring of patients at risk for inflammatory complications induced by trauma or infection.

## Abbreviations

CXCR-2, C-X-C motif chemokine receptor 2; NET, neutrophil extracellular trap; SI, systemic inflammation
